# Assessing Caregivers' Pre-visit Information-Seeking Behaviors for Pediatric Emergency Care: A Pilot Questionnaire Study in a University Hospital

**DOI:** 10.7759/cureus.107171

**Published:** 2026-04-16

**Authors:** Kei Narita, Naho Hirai, Rie Sato, Yoshiaki Iwashita

**Affiliations:** 1 Department of Emergency Medicine, U.S. Naval Hospital Okinawa, Okinawa, JPN; 2 Department of Emergency and Critical Care Medicine, Shimane University, Izumo, JPN

**Keywords:** consultation services, emergency department, health education, information seeking, non-severe illness, parental perception, pediatric

## Abstract

Background and objectives

Multiple informational resources - including online tools and the pediatric telephone consultation service (#8000) - are available in Japan to assist caregivers in determining whether to seek emergency care for their child. However, the utilization of these resources prior to pediatric emergency department (ED) visits remains unclear. This study aimed to examine whether caregivers sought information before visiting a pediatric ED and to identify which resources they utilized.

Materials and methods

We conducted a descriptive, questionnaire-based study involving caregivers who brought their child to the ED at a tertiary care center in a regional city in Japan between May and August 2022. Both paper-based and online questionnaires were used, consisting of 17 items that addressed patient demographics, pre-visit information searches, search methods, and clinical outcomes.

Results

Of the 42 distributed questionnaires, 36 valid responses were analyzed, resulting in a response rate of 85.7%. Among the 36 respondents, 21 caregivers reported seeking information prior to visiting the ED. The internet was the most frequently utilized resource, with 15 respondents indicating its use. Awareness of the #8000 hotline was high; however, only a minority utilized it, with just two respondents indicating usage. The perceived appropriateness of the visit did not differ significantly between caregivers who sought information and those who did not, with a p-value of 0.8.

Conclusions

These preliminary findings indicate that, although caregivers are aware of public information resources, many primarily rely on online searches before seeking emergency care. Further research, involving larger studies across more diverse settings, is necessary to elucidate how caregivers utilize these resources and how they can be better supported in their decision-making processes.

## Introduction

Emergency departments (EDs) in Japan have increasingly encountered overcrowding while receiving a significant number of patients with minor illnesses [1,2]. To alleviate the potential strain on emergency medical services, both national and local governments have implemented various measures to promote appropriate utilization of EDs. These measures include the nationwide pediatric telephone consultation service (#8000), which has been available since 2010, and a pediatric emergency care handbook distributed by local governments that offers guidance on how to address common symptoms [3]. Despite the availability of these resources, the extent to which caregivers use them prior to visiting a pediatric ED remains unclear.

Prior research in Japan has examined caregivers’ help-seeking behaviors in settings outside of the ED. For example, Inokuchi et al. investigated pre- and post-visit behaviors among users of an after-hours house call service, highlighting how individuals seek and use health information surrounding clinical encounters [4]. More broadly, international studies have demonstrated that caregivers frequently rely on online information rather than official consultation tools, indicating a global challenge in promoting the effective use of reliable health information [5]. Understanding how caregivers interpret and engage with these resources prior to deciding to visit the ED is essential for optimizing pediatric emergency care.

However, despite the availability of numerous advisory resources, little is known about how caregivers actually seek and use health information before presenting to an ED in Japan. This lack of evidence represents an important research gap in efforts to promote the appropriate use of pediatric emergency services. Therefore, this study aimed to (1) ascertain whether caregivers sought information before visiting the ED and (2) identify the resources they utilized.

Shimane University Hospital serves approximately 170,000 residents of Izumo City, the second-largest city in Shimane Prefecture, Japan. The pediatric population, defined as individuals aged 0 to 14 years, is estimated to be around 23,000 [6]. The hospital provides pediatric emergency care during daytime hours, after hours, and on holidays. Additionally, the region is served by a prefectural hospital that operates a similar system, as well as a nighttime emergency clinic that remains open until 2100 hours. The ED receives approximately 12,300 emergency patients and 2,500 ambulance transports annually, including about 1,200 pediatric emergency visits [7].

## Materials and methods

This descriptive, questionnaire-based study was conducted in the ED of Shimane University Hospital, a regional tertiary care center located in Izumo, Japan. Caregivers of pediatric patients aged 0 to 15 years who visited the ED between May and August 2022 were eligible for inclusion. Caregivers of patients suspected of having COVID-19 were excluded in accordance with institutional protocol. This study was designed as a small-scale, descriptive pilot conducted over a predefined, short study period.

No proprietary classifications, staging systems, scales, or scoring systems requiring permission or a license were used in this study. The questionnaire was adapted from a previously published survey, modified for local use, and the source has been cited accordingly [8]. Although the original instrument had not undergone formal validation, its structure and major item categories were preserved. The questionnaire was modified for local use and comprised 17 questions, primarily multiple-choice items. These included inquiries about the child's age, the relationship between the child and the respondent, symptoms, reasons for the emergency room visit, methods used to gather information prior to the visit, awareness of the #8000 service, outcomes, and post-visit impressions (Table 1).

**Table 1:** Questions included in the questionnaire *Multiple answers were allowed.

Questions
How old is your child?
What is your relation to the child?
What were the symptoms of your child? *
What was the reason for your visit to the ER?
Did you think of searching for or consulting on your child’s symptoms before visiting the ER?
How long did you search for the said information?
Did you get the information you wanted?
If you did not find the desired information, what do you think was the reason for it? *
What tool/s did you use for your search? *
If you used the internet for your search, which website/s did you visit? *
Have you ever heard about the Shimane Children's Emergency Handbook?
Have you ever heard about the #8000 service?
Does your child visit medical institutions regularly?
Which hospital/s does the child visit regularly? *
Are there any primary care physicians you consult regarding your child’s health?
Did your child get admitted to the hospital for this visit?
Do you think the timing of your visit to the ER was appropriate?
Why do you think so?
Are there any requests upon deciding whether or not to visit the ER?

Questionnaires were distributed sequentially to all eligible caregivers during operational hours by the administrative staff at the ED reception desk. Caregivers were requested to complete the questionnaire during or after their visit. An online version of the questionnaire was also created using Google Forms, with a QR code printed on physical copies to facilitate completion after the ED visit, if necessary. Respondents were provided with the option to complete the questionnaire either online or on paper.

Only complete questionnaires were used for analysis, and no data imputation was conducted. All variables, with the exception of age, were recorded as categorical responses. Free-text comments were excluded from the statistical analysis. Hospital admission was the sole available outcome indicator. The sample size represented the number of responses collected during the predefined study period. No a priori sample size calculation was performed, as the study was exploratory in nature.

Descriptive statistics were used to summarize participant characteristics and information-seeking behaviors. Categorical variables were compared using Fisher’s exact test, with a significance level set at p < 0.05. The statistical analyses were conducted using Stata software (version 16.1; StataCorp LLC, College Station, TX, USA). No multivariable analyses were performed, as the study lacked sufficient power to detect associations. A large language model was partially employed to assist in the writing of this manuscript.

The study received approval from the Medical Research Ethics Committee of the Shimane University Faculty of Medicine (approval no. 5945). Informed consent was obtained from all participants, and no personal identifiers were collected.

## Results

Overall, 42 questionnaires were distributed, and 37 responses were received. After excluding those with incomplete responses, 36 questionnaires were analyzed. All responses were submitted on paper, and no respondents utilized the online questionnaire. The patients' ages ranged from 0 to 15 years, with a median age of five years. Twenty-seven questionnaires were completed by mothers, while nine were completed by fathers. The most frequently reported symptoms included injury (13 patients), fever (six patients), and cough (six patients); 57% (17 patients) were brought to the ED due to parental concern. The background and characteristics of the respondents and patients are presented in Table 2.

**Table 2:** Background and characteristics of the respondents and patients *Multiple answers were allowed. ED: emergency department

Variable	n (%)
Relationship with the patient	
Mother	27 (75%)
Father	9 (25%)
Symptoms*	
Fever	6
Cough	6
Diarrhea	1
Vomit	1
Stomach ache	4
Headache	2
Wheezes	2
Dermatologic concerns	2
Injury	13
Optic concerns	6
Others	5
Reason for visits to the ED	
Child’s condition seemed severe	2 (6%)
Symptoms exacerbated	4 (11%)
Caregivers were worried	17 (47%)
Unable to visit during open hours	2 (6%)
Recommended to visit the ED	4 (11%)
Wanted prescribed medications	1 (3%)
Regular visit to the hospital	
Patient regularly visited the hospital	27 (75%)
Prefecture Central Hospital	2
Shimane University Hospital	10
Another hospital with an ED	1
Another hospital without an ED	20
Others	2
No regular visits	9 (25%)
Access to the primary care physician	
Available	32 (89%)
Not available	2 (6%)
Unanswered	2 (6%)
Hospital admission after this visit	
Admitted	0 (0%)
Not admitted	36 (100%)

Information search

We found that 58.3% (n = 21) of the respondents sought information regarding their children’s symptoms prior to visiting the ED, whereas 41.7% (n = 15) did not. Among those who sought information, the internet was the most frequently used method (15 of 36; Table 3). Only two respondents utilized the #8000 service as a source of information.

**Table 3:** Information search regarding patient symptoms by the respondents

Was an information search performed before the emergency department visit?	Respondents
Yes	21 (58.3%)
No	15 (41.7%)

Were the respondents able to gather the required information?

We also inquired whether participants were able to obtain the information they sought. A total of 85.7% of respondents (18 out of 21 participants who searched for information prior to their ED visit) reported successfully locating the desired information. Among the 15 individuals who utilized the Internet for their search, nine were able to find the information they were looking for, whereas six were not. Additionally, four out of five participants who consulted family and friends successfully gathered the desired information (Table 4).

**Table 4:** Tools used for the information search and successful information collection *Included calling the university hospital, consulting other medical staff, and consulting the home-visiting nursing service.

Tools used to search for the symptoms		Total (n)	Obtained the desired information	Could not obtain information
Internet	15		9 (60%)	6 (40%)
Books	0		N/A	N/A
Child’s Emergency Booklet (Shimane)	0		N/A	N/A
#8000 telephone triage service	2		1 (50%)	1 (50%)
Primary care physician	3		1 (33.3%)	2 (66.6%)
Consulted family and friends	5		4 (80%)	1 (20%)
Others*	3		3 (100%)	0 (0%)

Did the respondents know about handbooks and the #8000 service?

Half of the respondents had previously used the #8000 telephone service, and 22.2% were aware of the service but had never used it. Additionally, 11.1% had only heard of the service's name, and 15.8% had never heard of it (Table 5). Two respondents had used the #8000 service prior to visiting the ED; however, only one indicated that they were able to access the desired information through the service. Furthermore, only one respondent had previously utilized the handbook, and none had used it to assess their children’s symptoms before visiting the ED.

**Table 5:** Awareness and use of two child-related information services

	#8000 telephone service	Child’s emergency booklet, Shimane
Have used the service	18 (50%)	1 (2.8%)
Know about it, but never used it	8 (22.2%)	2 (5.3%)
Heard only the name	4 (11.1%)	4 (11.1%)
Never heard of the service	6 (15.8%)	29 (80.6%)

Self-evaluation of whether the ED visit was appropriate

**Figure 1:**
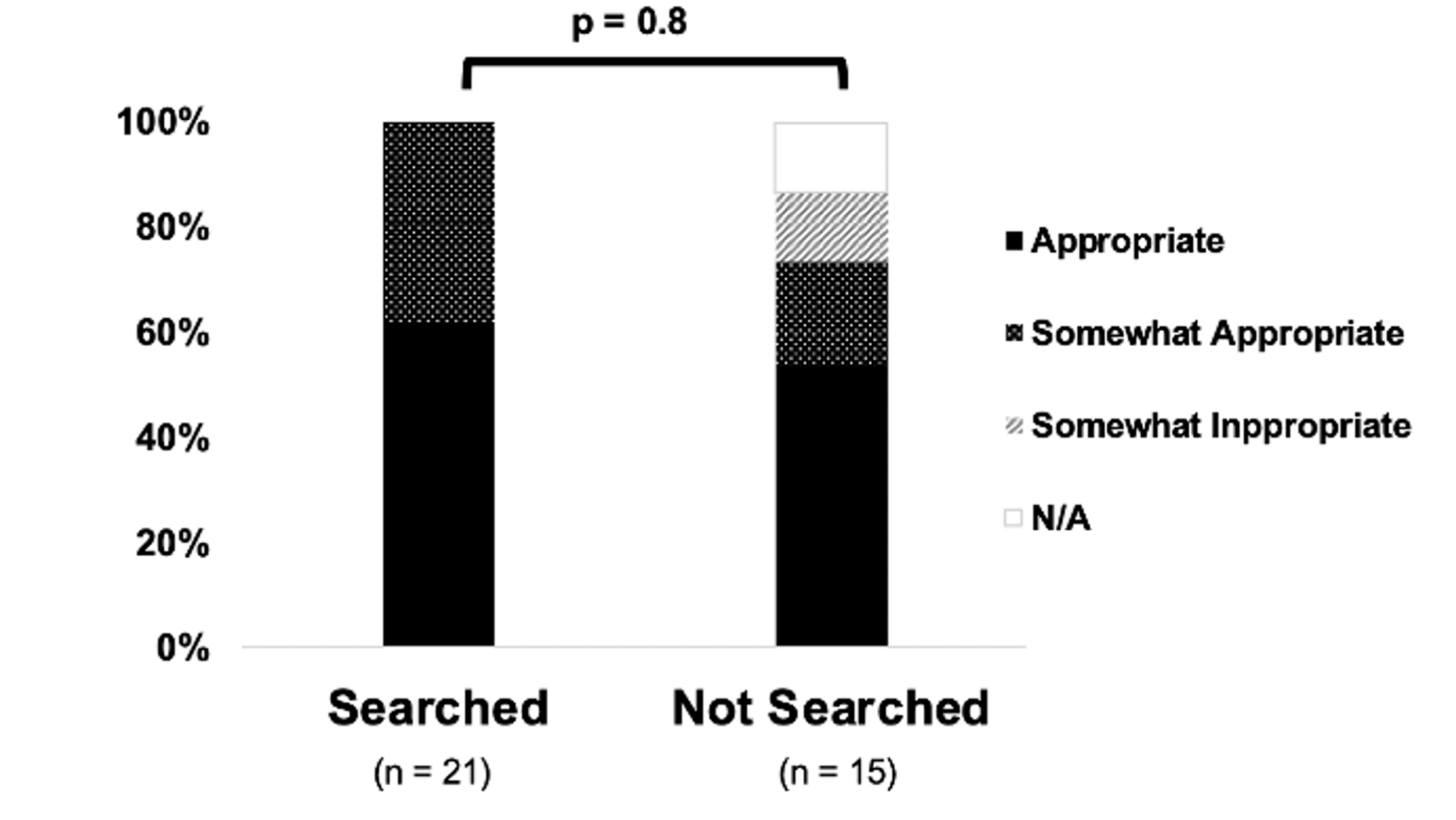
Self-evaluation of the appropriateness of the ED visit The respondents were asked to rate the appropriateness of the timing of their ED visit. Responses were collected and analyzed to determine whether conducting research before visiting the ED would have resulted in different responses. ED: emergency department

We compared the search and no-search groups to assess any differences in their self-evaluations of ED visits. Participants were asked to rate their ED visits as appropriate, somewhat appropriate, somewhat inappropriate, or inappropriate. No significant differences in how respondents rated their ED visits were observed, regardless of whether they had searched for information before visiting the ED (p = 0.8; Figure 1).

## Discussion

This study examined the information-seeking behaviors of caregivers who visited a pediatric ED in a regional city in Japan. Approximately half of the caregivers conducted information searches prior to their visit, with most relying on online resources. Although many respondents were aware of government-provided resources, only a subset obtained information from these sources that influenced their decision-making. These findings indicate a gap between awareness of available tools and their practical application. Additionally, the perceived appropriateness of the ED visit did not show a significant difference between caregivers who sought information and those who did not.

Our findings are consistent with previous studies indicating that caregivers frequently depend on online resources when assessing their child’s symptoms. Wong and Cheung identified that 87.4% of patients who visit primary care clinics had used the internet to find health information [9], while Khoo et al. found that 43% had used the internet within the previous six months, but only 6% used it immediately prior to the ED visit of the children [10]. Sarria-Guerrero et al. proposed that triage tools could potentially decrease non-urgent ED visits [11]. Although the settings vary, the pattern observed in this study - awareness without consistent utilization - aligns broadly with these trends. Nonetheless, owing to the limited sample size, these interpretations should be regarded as preliminary. The discrepancy between caregivers' high awareness of the #8000 service and their limited actual usage indicates a persistent utilization gap, the reasons for which remain unclear based on the current data. Previous research in Japan has suggested that parental anxiety regarding the accessibility and reliability of emergency medical service systems may contribute to this hesitation [12].

For official information sources to function effectively, three steps are necessary: first, caregivers must know in advance that such resources exist; second, they must actually use them; and third, they must act on the basis of the information obtained. A previous international systematic review reported that, although many parents experience substantial anxiety and desire more detailed guidance from pediatricians, they rarely discuss which information sources should be considered trustworthy in everyday practice [13]. In our study, many caregivers were aware of the #8000 service but did not use it. In the study by Sarria-Guerrero et al., telephone consultation was used to recommend ED visits for children judged to have high-acuity conditions; although 57.4% of caregivers had already intended to visit the ED before the call, 46% reported that they changed their intention after the telephone consultation [11].

Taken together, these findings suggest that future pediatric emergency care systems should include not only education - centered on pediatricians - about which information resources should be used during emergencies, but also mechanisms that improve access during actual emergencies and reduce caregiver anxiety through appropriate information provision. In the survey conducted by Drent et al., the dissemination of health information by healthcare professionals through multiple modalities was also considered the most effective approach [14].

This study achieved a relatively high response rate and provided preliminary descriptive data on caregivers' information-seeking behavior in this region. These findings may inform the design of future investigations. Furthermore, caregivers completed the questionnaire immediately after their ED visit, which likely reduced recall bias and accurately captured their pre-visit behaviors. Assessing both awareness and real-world utilization also offers insights into the gap between the availability of information resources and actual caregiver decision-making.

This study has a few limitations. The sample size was notably small and was derived from a single regional hospital over a restricted timeframe, which constrains the generalizability of the findings. Additionally, the survey included only caregivers who presented to the ED, rendering selection bias inevitable; thus, the information-seeking behaviors of caregivers who opted not to pursue emergency care remain unexamined. Furthermore, the questionnaire lacked formal validation, which may compromise the accuracy of certain measurements. Hospital admission was the sole available outcome indicator and should not be interpreted as a proxy for disease severity; consequently, the current findings cannot ascertain whether information-seeking behavior varied based on illness acuity. Lastly, follow-up information regarding subsequent caregiving decisions was not collected. Despite these limitations, the findings underscore potential areas for improving caregiver support. Enhancing the accessibility and clarity of available informational resources may assist caregivers in making more informed decisions prior to seeking emergency care.

Future studies should extend beyond a single regional setting and include variables not captured in the current survey, such as socioeconomic status and health literacy [15], which may influence caregivers’ information-seeking behaviors. Employing validated questionnaires and integrating qualitative approaches may help identify how caregivers interpret available resources and why public services, such as #8000, remain underutilized. Furthermore, follow-up assessments could clarify how caregivers use information after the ED visit and how this impacts subsequent decision-making. Additionally, comparative research evaluating traditional consultation services alongside emerging digital resources - such as AI-supported triage systems - may help determine which formats most effectively support timely and appropriate ED use. For instance, recent studies on mobile health applications in Japan have shown promise in improving parental decision-making regarding emergency visits [16]. These avenues will be crucial for developing more effective caregiver support strategies and enhancing pediatric emergency care delivery.

## Conclusions

These preliminary findings provide a foundation for understanding caregiver information-seeking behavior in a regional Japanese context. Although awareness of public resources, such as the #8000 telephone service, was notably high among the study participants, actual utilization remained limited, underscoring a persistent gap between the availability of official information tools and their real-world application. Addressing the barriers that prevent caregivers from engaging with these resources - whether related to accessibility, trust, or usability - may help promote more informed and appropriate use of pediatric emergency care. Further research, with larger and more diverse samples, will be necessary to elucidate how caregivers utilize available resources and how these tools can be enhanced to better support parental decision-making.

## References

[1] (2025). Shimane Prefecture Health and Medical Care Plan (in Japanese). https://www.pref.shimane.lg.jp/medical/kenko/iryo/shimaneno_iryo/hokenniryoukeikaku/index.data/15_shoniiryo.pdf.

[2] Morley C, Unwin M, Peterson GM, Stankovich J, Kinsman L (2018). Emergency department crowding: a systematic review of causes, consequences and solutions. PLoS One.

[3] (2025). Shimane Children’s Emergency Handbook (in Japanese). https://www.pref.shimane.lg.jp/medical/kenko/iryo/shimaneno_iryo/kodomo.html.

[4] Inokuchi R, Morita K, Jin X, Ishikawa M, Tamiya N (2021). Pre- and post-home visit behaviors after using after-hours house call (AHHC) medical services: a questionnaire-based survey in Tokyo, Japan. BMC Emerg Med.

[5] Shroff PL, Hayes RW, Padmanabhan P, Stevenson MD (2017). Internet usage by parents prior to seeking care at a pediatric emergency department: observational study. Interact J Med Res.

[6] (2025). Izumo City. Population by age as of 30 August 2025 (in Japanese). https://www.city.izumo.shimane.jp/www/contents/1184806835555/files/2508JNNEP.pdf.

[7] (2025). Survey of pediatric (secondary/tertiary) emergency medical service provision systems - summary tables (FY 2020) (in Japanese). https://www.mhlw.go.jp/content/10800000/001151253.pdf.

[8] Kusano J, Takano M, Fujita Y (2015). Internet usage and care-seeking decisions among parents visiting pediatric emergency outpatient services (in Japanese). J Nurs Sci Res.

[9] Wong DK, Cheung MK (2019). Online health information seeking and eHealth literacy among patients attending a primary care clinic in Hong Kong: a cross‑sectional survey. J Med Internet Res.

[10] Khoo K, Bolt P, Babl FE, Jury S, Goldman RD (2008). Health information seeking by parents in the Internet age. J Paediatr Child Health.

[11] Sarria-Guerrero JA, Luaces-Cubells C, Jiménez-Fàbrega FX, Villamor-Ordozgoiti A, Isla Pera P, Guix-Comellas EM (2019). Pediatric televisits and telephone triage: impact on use of a hospital emergency department. Emergencias.

[12] Sobue I, Tanimoto K, Itoh S (2017). A scale of parental anxiety about pediatric emergency medical care services of Japan: development, reliability, validity, generalizability and usefulness. Health.

[13] Kubb C, Foran HM (2020). Online health information seeking by parents for their children: systematic review and agenda for further research. J Med Internet Res.

[14] Drent AM, Brousseau DC, Morrison AK (2018). Health information preferences of parents in a pediatric emergency department. Clin Pediatr (Phila).

[15] Schillinger D, Bindman A, Wang F, Stewart A, Piette J (2004). Functional health literacy and the quality of physician-patient communication among diabetes patients. Patient Educ Couns.

[16] Sakamoto M, Suzuki A, Ishikawa H (2025). Association between the use of an app for providing healthcare information for parents and urgent emergency department visits for children: a cross-sectional study in Japan. BMJ Open.

